# Characterization of the repertoire diversity of the *Plasmodium falciparum stevor *multigene family in laboratory and field isolates

**DOI:** 10.1186/1475-2875-8-140

**Published:** 2009-06-26

**Authors:** Jane E Blythe, Makhtar Niang, Kevin Marsh, Anthony A Holder, Jean Langhorne, Peter R Preiser

**Affiliations:** 1 Nanyang Technological University, School of Biological Sciences, 60 Nanyang Drive, Singapore 637551, Singapore; 2 Division of Parasitology, National Institute for Medical Research, The Ridgeway, London NW7 1AA, UK; 3 Kenya Medical Research Institute (KEMRI), PO Box 230, Kilifi, Kenya

## Abstract

**Background:**

The evasion of host immune response by the human malaria parasite *Plasmodium falciparum *has been linked to expression of a range of variable antigens on the infected erythrocyte surface. Several genes are potentially involved in this process with the *var*, *rif *and *stevor *multigene families being the most likely candidates and coding for rapidly evolving proteins. The high sequence diversity of proteins encoded by these gene families may have evolved as an immune evasion strategy that enables the parasite to establish long lasting chronic infections. Previous findings have shown that the hypervariable region (HVR) of STEVOR has significant sequence diversity both within as well as across different *P. falciparum *lines. However, these studies did not address whether or not there are ancestral *stevor *that can be found in different parasites.

**Methods:**

DNA and RNA sequences analysis as well as phylogenetic approaches were used to analyse the *stevor *sequence repertoire and diversity in laboratory lines and Kilifi (Kenya) fresh isolates.

**Results:**

Conserved *stevor *genes were identified in different *P. falciparum *isolates from different global locations. Consistent with previous studies, the HVR of the *stevor *gene family was found to be highly divergent both within and between isolates. Importantly phylogenetic analysis shows some clustering of *stevor *sequences both within a single parasite clone as well as across different parasite isolates.

**Conclusion:**

This indicates that the ancestral *P. falciparum *parasite genome already contained multiple *stevor *genes that have subsequently diversified further within the different *P. falciparum *populations. It also confirms that STEVOR is under strong selection pressure.

## Background

*Plasmodium *malaria is an infectious disease of global significance with more than 500 million people suffering from the disease and at least one million dying each year [1]. The *P. falciparum *genome project [2] has revealed the presence of several multicopy gene families which code for hypervariable antigens that are exported to the surface of the infected host erythrocyte and represent targets of naturally acquired immunity to malaria. To date *var*, *rif *as well as *stevor *have been described [2-4] as the major variant surface antigens (VSAs). These multigene families are predominantly situated at the sub-telomeric ends of chromosomes [2,5,6], where gene rearrangements are frequent [7,8] leading to high rates of recombination, thus facilitating their rapid evolution and diversity.

While some *rif *and *var *genes are found in centrally located clusters, most *stevor *genes are found at chromosome ends [2]. The high sequence diversity of proteins encoded by these gene families may have evolved as an immune evasion strategy that enables the parasite to establish long lasting chronic infections [9].

The *P. falciparum *genome contains between 50 and 60 *var *genes [2] that code for the Erythrocyte Membrane Protein 1 (PfEMP1) [10-12]. EMP1 is linked not only to immune evasion but also to parasite sequestration and pathology and is by far the best studied multigene family in *P. falciparum *[11,13,14].

*Stevor *and *rif *share structural similarities and this relationship is emphasized by the existence of a RIFIN-STEVOR family (PF02009) in the PFAM database [15]. Characteristically, they are defined as small polypeptides with a putative signal sequence followed by a semi-conserved domain, an HVR and a conserved C-terminal domain [16]. In both protein families, two predicted transmembrane-spanning regions flank the HVR which has been predicted to form a loop on the infected red blood cell (iRBC) surface, exposing it to the host immune system and probably resulting in distinct evolution into novel functional units [3,16]. However, STEVOR and RIFIN differ in that the HVR of RIFIN is up to 300 bp longer than the equivalent region in STEVOR. Moreover, STEVOR are a distinct, more conserved, and lower copy number family [17] than RIFIN. The *rif *and *stevor *(150–200 copies and 30–33 copies respectively in the 3D7 genome) repertoire diversity has been studied in the 3D7 parasite clone, and in several other laboratory parasite lines from different geographic origins [18,19]. Based on these studies, it is clear that the HVR of STEVOR has significant sequence diversity both within as well as across different *P. falciparum *lines. These studies did not address whether or not there are ancestral *stevor *that can be found in different parasites.

The entire genome of the *P. falciparum *3D7 parasite clone was sequenced completely first as part of the malaria genome project [2]. A number of additional lines have now also been at least partially sequenced so that there is now considerable information on the VSA repertoire on these parasite species. The ancestors of parasites isolated worldwide are most likely to be from Africa [20]. A study of *var *gene sequences in 12 Kilifi parasite isolates has shown a comparable number of broadly classified types as the 3D7 genome [21]. Similarly, another study of *var *sequences from Amazonian parasite isolates indicated that the expected gene repertoire was again similar to that of the 3D7 genome and that the Amazonian *var *genes were most similar to *var *identified in other American isolates [18].

The 3D7 *var *genes have been grouped into three majors types based on sequence analysis of the intron and 5' and 3' un-translated regions (UTR) [2,22,23], and the Kilifi *var *had a similar type distribution [21]. Recently, phylogenetic approaches have enabled subdivision of the RIFIN protein family into two major A- and B-subgroups [24] associated with distinct subcellular location and developmental expression patterns [25].

Such important data are currently missing for the *stevor *multigene family and it is not known whether field isolates contain a similar number of *stevor *to that of the 3D7 clone. In addition, a more extensive study of *stevor *from isolates worldwide is required in order to estimate both the repertoire size and the extent of diversity. Comparative analysis of long-term laboratory-adapted parasites and fresh clinical isolates has shown that laboratory-adapted lines are not significantly more or less divergent than recent isolates [26]. However, the 3D7 clone was shown to have accumulated DNA in culture through insertions of +1nucleotide/kb [27], and this might suggest divergent sequences compared to fresh isolates.

In this study, DNA and RNA sequence analysis were combined as well as phylogenetic approaches to analyse the *stevor *sequence repertoire and diversity in laboratory lines and fresh isolates from Kilifi in Kenya. Conserved *stevor *genes (orthologues) were identified in different *P. falciparum *isolates from different global locations. Consistent with previous studies, the HVR of the *stevor *gene family was found to be highly divergent both within and between isolates. Importantly, phylogenetic analysis shows some clustering of *stevor *sequences both within a single parasite clone as well as across different parasite isolates. These data indicate that the ancestral *P. falciparum *parasite genome already contained multiple *stevor *genes that subsequently diversified further within the different *P. falciparum *populations.

## Methods

### Parasites

Parasites used in this study were either well-characterized laboratory lines or were isolated from blood samples obtained at the KEMRI-Wellcome Trust, Kilifi, Kenya. Parasite lines and their origin are listed (Table 1). The African parasites were collected at Kilifi district hospital, situated 60 km north of Mombasa on the Kenyan coast. More than 10% of the children under five years of age, who reside within the Kilifi district, are admitted into hospital each year. In this area, *P. falciparum *transmitted by mosquitoes of the *Anopheles gambiae *complex [28], is seasonal and usually occurs after the long and short rainy seasons.

**Table 1:** Origin of *P. falciparum *clones used for *in vitro *culture

**Clone**	**Parent isolate**	**Geographical origin**	**Reference**
3D7	NF54	Amsterdam airport -Netherlands (unknown origin)	(Walliker *et al*., 1987)
A4	IT04	Brazil	(Roberts et al., 1992)
C10	1776	Malaysia	(Ang *et al*., 1996)
D10	FCQ-27	Papua New Guinea	(Chen *et al*., 1980)
Dd2	W2-MEF	Indochina	(Oduola *et al*., 1988)
FcB1	Unknown	Colombia	(Schrevel *et al*., 1994)
T9/96	T9	Thailand	(Thaithong *et al*., 1984)

This study was approved by the Institutional Ethical Review Board of Nanyang Technological University, Singapore, and by the Kenyan Medical Research Institute National Ethics Committee

### Culture of *P. falciparum *laboratory lines and Kilifi isolates

Laboratory parasite lines were cultured in fresh human RBC (supplied by National UK Blood Donation Service) in RPMI 1640 complete medium supplemented with Albumax and 2 mM glutamine (Gibco) as previously described [29]. Parasitaemia was monitored daily by microscopy examination of Giemsa stained thin blood smears.

*Plasmodium falciparum *parasite isolates from Kilifi were recovered from liquid nitrogen frozen stock and cultured in RPMI 1640 medium supplemented with 10% AB human serum (Kilifi General Hospital, Kenya), 2 mM L-glutamine (Gibco), 37.5 mM HEPES (Gibco), 20 mM Glucose, and 2 μg/ml Gentamycin (Gibco). No fresh red blood cells (RBCs) were added and the parasites were maintained in culture for approximately 30 hours, or until they reached late trophozoite/schizont-stages. Parasite samples were separated into two fractions for later processing of parasite genomic DNA (gDNA) and RNA isolation.

### Preparation of parasite genomic DNA (gDNA) and RNA

*Plasmodium falciparum *iRBCs were pelleted at 560× *g *for 5 minutes. Resulting pellets were stored at -80°C in screw top cryovials. A phenol/chloroform extraction method was used for larger (greater than 500 μl) iRBC pellets as previously described [30]. The DNeasy^® ^kit (Qiagen) protocols 1 and 2 were used for small (less than 500 μl) blood samples according to manufacturer's recommendations. Genomic DNA was resuspended in nuclease-free water.

For RNA isolation, iRBC pellets (approximately 100 μl) were lysed in 1 ml prewarmed (37°C) Trizol LS reagent (Gibco Invitrogen) and then stored at -80°C in screw top cryovials. RNA was extracted as described previously [31], using DNase I digestion (Invitrogen) to remove contaminating DNA. RNA was resuspended in nuclease-free water.

### Primers designed for *stevor *specific PCR/RT-PCR

The primers were assessed using multiple sequence alignments to ensure that the chosen sequences were in regions conserved within the *stevor *gene family, but not in other genes (for example the *rif *gene family). A combination of two sets of primers in a nested PCR was used RepF1, RepF2 and RepR internal-PCR primers were designed as previously described [16]. The RepF1/2 primers were redesigned to remove an opal stop codon (TGA) replacing it with (TGC). The smf1/smr1 external primers were previously described [32]. All primers were synthesized by Eurogentec/Oswel (Southampton, UK) or Research Biolabs (Singapore). The use of a nested-PCR protocol for RT-PCR was problematic due to amplification of contaminating gDNA, therefore a *stevor*-specific single step amplification was designed, facilitated by the complete genome sequence [2], using the primers JBSTEVORF1 and JBSTEVORR1. Primers sequences are summarized in Table 2.

**Table 2:** Primers sequences

**Primer name**	**Direction**	**Primer Sequence (5'-3')**	**Tm (°C)**
Smf1	sense	GAYSCAGAACTCAARGAAATWATT	46.42
Smr1	antisense	GCAGMACCAAAGYWGYAATACC	47.9
JBSTEVORF1	sense	AGATGTACCCGTGGTATATGTTCTTGCA	54.96
JBSTEVORR1	antisense	GCTAATATAAGTAGAACCAAAGCTGCAATAC	54.44
RepF1	sense	AGATGAATTCGTGGTATATRTTYTTGYTC	55.84
RepF2	sense	AGATGAATTCGGGGTTTAGTTGTGTGTGC	63.86
RepR	antisense	TTTAGGATCCAGAACCAAARYTGCAATACC	62.21

DNA and RNA products were separated by electrophoresis in 1% multi purpose agarose (Roche) in 1× Tris buffer contained 0.25 μg/ml ethidium bromide to enable UV visualization. RNA gels contained 5 mM guanidine thiocyanate.

### Small-scale preparation of plasmid DNA (minipreps)

Plasmid DNA was isolated using the QIAprep spin SNAP Miniprep^® ^Kit (Invitrogen). The QIAgen Maxiprep^® ^kit was used according to manufacturer's protocol to isolate larger quantities of pET-24a (+) vector.

### Automated DNA Sequencing

Following DNA expansion, purification, and confirmation that a *stevor *insert was present; the DNA was then quantified by spectrophotometry, pelleted and dried before sequencing.

Three systems have been used for sequencing reactions:

a) The BigDye^® ^Terminator Cycle Sequencing Ready Reaction kit (Perkin-Elmer, Applied Biosystems, Warrington, UK) was used according to manufacturer's instructions. The reaction was carried out using approximately 600 ng of pCR^®^2.1 or pCR^® ^II DNA (vector plus insert) per sample with either M13 forward or reverse primers in the Eppendorf Mastercycler. Unincorporated dye terminators were removed by ethanol precipitation. Samples were resuspended in 3 μl loading dye 16.5% v/v Blue Dextran/EDTA (Applied Biosystems) in formamide (Amresco, Solon USA), followed by electrophoresis and data collection on the ABI PRISM™ 377 DNA sequencer (Perkin -Elmer).

b) The BigDye^® ^reactions were also used with T7 primers and pET-24a (+) DNA, or TrxFus forward or T7 reverse primers with pET102/D-TOPO DNA. Sequencing was performed at the National Neuroscience Institute, Singapore.

c) The MegaBACE system (Amersham Biosciences) was used. The ABI PRISM^® ^BigDye^® ^Terminator v1.1 Cycle Sequencing kit (Applied Biosystems) cycle sequencing mix was used according to the manufacturer's protocols. Samples were ethanol precipitated before addition of MegaBACE™ loading solution (Amersham Biosciences) and loading onto a MegaBACE 96-capillary machine.

### Sequence alignment analysis (HVR region)

A fragment of approximately 280 base pairs (bp) was successfully isolated through PCR and RT-PCR amplification. However, due to the variant nature of the HVR, sequences varied in length, coding for a maximum of 82 amino acids. Clones have been obtained from multiple PCR reactions to reduce PCR bias, and were sequenced in forward and reverse directions. *P. falciparum *3D7, IT and Ghanaian isolates sequences were obtained from BLAST and Tools-oligo searches within PlasmoDB [33].

Editing of nucleotide and amino acid (translated) sequences was carried out using the Autoassembler^®^, Chromas^®^, DNAstar (2001) freeware programmes. Sequences from different parasite sources and different amplification reactions were aligned by using ClustalX^® ^[34], and Bioedit7.0.1^® ^softwares. Multiple alignments and their corresponding chromatograms were edited by eye for discrepancies and mistaken nucleotide assignment by the automated sequencing method. BLAST analysis, version 4.4 from PlasmoDB [33] was used in order to confirm that DNA sequences were the result of the amplification of *stevor *exon-2 hyper-variable loop locus.

### Phylogenetic analysis

Phylogenetic trees showing genetic distance were constructed using the following programmes: Molecular Evolutionary Genetics Analysis (MEGA) 2.1 or 3.0^®^, PAUP version 4.0 [35,36], and Tree view^® ^[37]. These programmes enable the construction of trees using three-building models: Neighbor-Joining, Maximum Parsimony and Maximum likelihood, taking into account the observed nucleotide frequency, substitution rate and transition/transversion ratio, based on Nei genetic distance estimates [35]. The bootstrap analysis [38] was used in order to statistically assign support to hypotheses of phylogenetic relationship among sequences estimated by the above-mentioned methods.

## Results

### Identification of common *stevor *genes in laboratory lines and Kilifi field isolates

The *P. falciparum *genome sequencing project [2] as well as subsequent sequencing efforts has given some insight into the diversity of *stevor *in different *P. falciparum *lines. Still these data are limited and, therefore, additional sequences from both established laboratory lines and freshly isolated parasite samples would provide valuable additional information. For this purpose, genomic DNA was extracted from five different laboratory lines of various geographic origins: A4; FcB1; C10; T9/96 and 3D7 (Table 1) and also from 10 representative blood samples taken from patients in the Kilifi region of Kenya.

**Figure 1:**
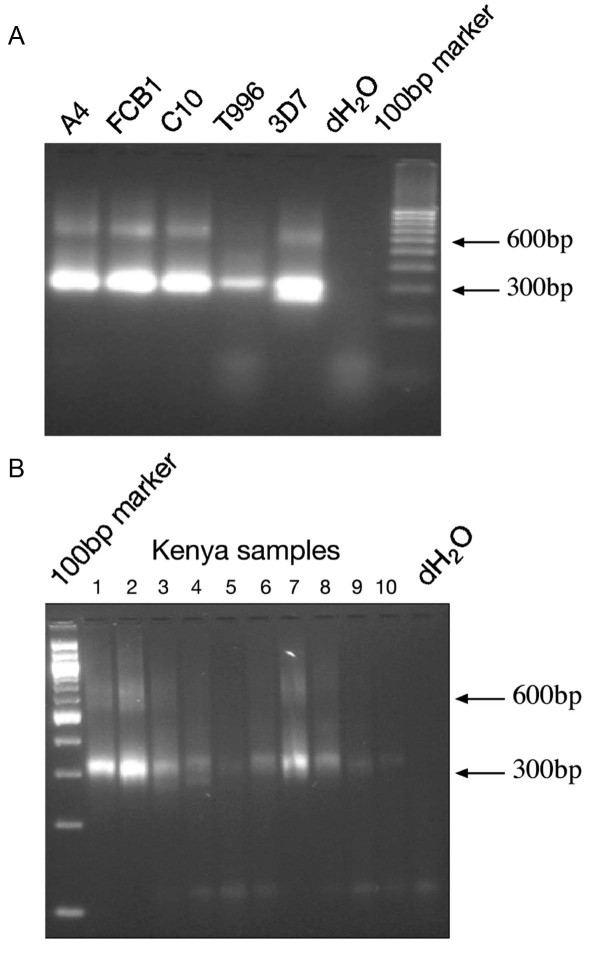
**Agarose gel showing PCR products obtained from laboratory line and Kilifi isolate parasite genomic DNA**. Gel electrophoresis of nested *stevor *PCR products from genomic DNA extracted from 5 representative *P. falciparum *laboratory lines: A4; FcB1; C10; T9/96; and 3D7 (A) and 10 representative Kilifi parasite isolates (B). 5 μl of each product was loaded onto a 2.5% agarose gel. Results show two amplicons in the expected size ranges, products carried over in the nested PCR-step from the external PCR (approximately 600 bp) with primers smf1 and smr1, and internal PCR products (approximately 300 bp) from primers RepF1/2 and RepR. 100 bp marker (Fermentas) is shown. dH_2_O = negative control containing sterile water instead of template DNA.

Genomic DNA was amplified using the previously described *stevor*-specific nested primers flanking the predicted hyper-variable loop region [32] (Table 2). PCR from all laboratory lines (Figure 1A) as well as Kilifi isolates (Figure 1B) gave amplification products of the expected size (600 bp and 300 bp) indicating that these primers were able to amplify *stevor *sequences from both the laboratory lines and Kilifi field isolates. Similar to what was found with the *P. falciparum *3D7, more than one PCR product was detected in the expected size range, amplified by both the external (550–700 bp), and internal primers (250–350 bp) (Figure 1A). This is indicative of the amplification of multiple *stevor *sequences from both laboratory lines and field isolates. The 300 bp PCR products from the different samples were cloned and a number of clones were sequenced in both forward and reverse directions. The sequence data obtained here from genomic DNA was further expanded by the addition of the transcribed *stevor *sequences which had previously been obtained by RT-PCR from RNA extracted from the same original blood samples [39].

**Figure 2:**
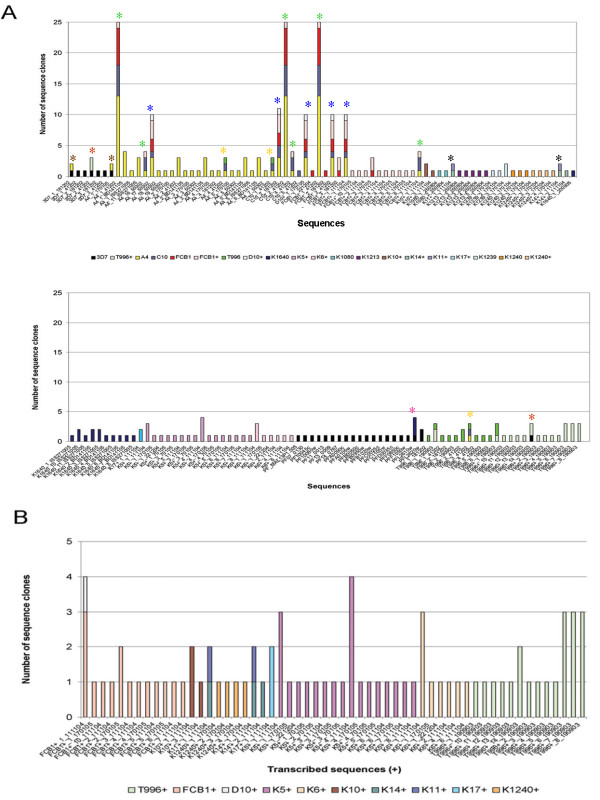
**Identification of *stevor *sequences per parasite isolate**. A) Number of different *stevor *obtained from parasite DNA and RNA. Parasite isolates are shown in different colors below the graph. The sequence name is denoted along the x-axis, each sequence has a unique identifier consisting of the parasite isolate (+ if amplified from RNA), clone number and date. The number of clones per sequence is shown on the y-axis. *P. falciparum *3D7 genomic *stevor *sequences are included for comparison (solid black). B) Representation of the different transcribed (+) *stevor *obtained in this study.

Despite the low throughput approach, a total of 213 genomic DNA and RNA products were successfully sequenced, 133 were unique DNA sequences [see Additional file 1] coding for 108 unique amino acid sequences. Comparison with known STEVOR sequences confirmed that the PCR and RT-PCR products indeed represented STEVOR. The number of times each sequence was obtained per isolate is shown (Figure 2A). Overall, six different *stevor *sequences were identified from genomic DNA in the reference 3D7 clone isolated in The Netherlands but of unknown geographic origin [40] that had been included as a positive control. Interestingly, two (3D7_2_41202 and 3D7_1_181202) of the sequences obtained from the 3D7 clone maintained at the NIMR in London were absent from the published 3D7 genome *stevor *repertoire (Figure 2A). All other *stevor *sequences present in the 3D7 genome are shown with their gene identification number for the purpose of comparison. Of the 133 unique *stevor *DNA sequences, twenty-one were found to be present in more than one parasite line or isolate (Figure 2A, asterisk), five (5) of these paralogues were identified in A4, C10, FcB1 and D10 (blue asterisk), six (6) were found in A4, C10 and FcB1 (green asterisk), three (3) were found in T9/96, C10 and A4 (orange asterisk), while two (2) were found in 3D7 and T9/96 (red asterisk), 3D7 and A4 (brown asterisk) as well as K11 and K14 (black asterisk). Finally a single sequence was found as part of the 3D7 genome and K1640 (Figure 2A, pink asterisk). The highly represented *stevor *sequences most likely represent "common" *stevor *that can be found in a variety of parasite isolates from around the world. For a number of these more common *stevor *transcription was also detected by RT-PCR indicating that these genes are of potential functional relevance.

From this initial analysis it is clear that there are a number of *stevor *sequences that are common to a number of different laboratory lines; in contrast only a single sequence (K1640_1_BD141105) from a Kilifi isolate was found to be identical to a gene (PFL2610w) from the 3D7 genome. In contrast despite the large number of *stevor *sequences identified in the Kilifi isolates, only a single *stevor *sequence was found to be present in more than one isolate. This particular sequence was transcribed in both K11 (K11+_111104) and K14 (K14+_1_111104) parasites (Figure 2B).

The RT-PCR data indicate that *stevor *is transcribed in all the *P. falciparum *lines examined with the exception of the parasite clone A4, supporting previous observations that this parasite clone does not transcribe nor express STEVOR [4,39]. This lack of STEVOR expression in the A4 parasite is not due to the absence of the genetic information as this clone contains at least 20 different *stevor *genes (Figure 2A). In line with published observations [21] that field isolates transcribe multiple *var *at the same time, numerous unique *stevor *transcripts were also detected in both laboratory and patient samples (Figure 2B).

### Conservation of *stevor *across different parasite lines

**Figure 3:**
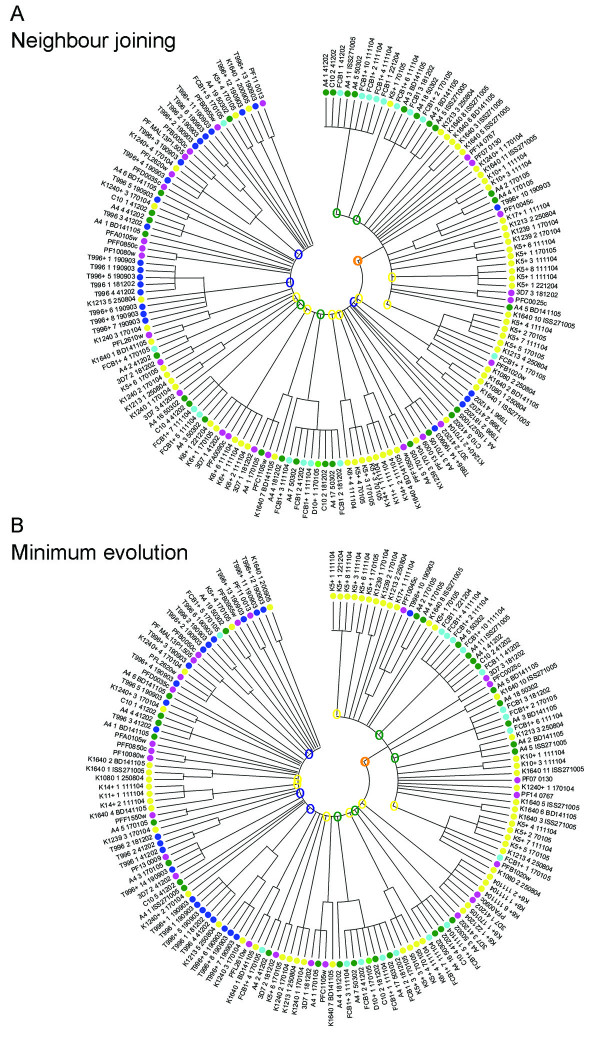
**Phylogenetic trees of *stevor *DNA sequences**. Neighbor-joining (A) and Minimum Evolution (B) methods were used for generation of phylogenetic trees of 152 HVR *stevor *alleles. Trees are based upon a nucleotide alignment of the HVRs and condensed to show only those branches with a bootstrap value of 95% significance and above. Sequences are color-coded according to originating isolate: Kilifi-yellow; T9/96-dark blue; 3D7-pink; A4 and C10-green; FcB1-light blue; D10-light green. Orange open-circle around a branch point indicates the major *stevor *sub-grouping, blue open-circles highlight T9/96 sub-groups, green open-circles highlight sub-groupings including FcB1, C10 and A4 sequences, yellow open-circles highlight Kilifi sequence sub-groupings.

The observation that identical HVR sequences could be found in different parasite lines raised the possibility that *stevor *could be grouped into distinct subgroups with different biological functions, similar to the *rif *and *pir *multigene family [25,41,42]. For this purpose the sequences identified here, together with the corresponding regions from the 3D7 *stevor *repertoire were clustered into similarity groups by constructing a Neighbor Joining as well as a Minimum Evolution Tree (Figure 3).

The Neighbor Joining tree (Figure 3A) clustered the 152 HVR DNA sequences into two groups with one large sub-group containing approximately one-third of the *stevor *sequences (Figure 3A, orange branch). This subgroup contained sequences from all parasites except the D10 laboratory line and the K14, K11 and K6 Kilifi field isolates. At this stage it is not clear whether the absence of this subgroup of *stevor *in these parasites is a reflection of the limited sequence data available or truly represents a lack of this subgroup. It is also interesting to note that there is further sub-clustering of sequences within this branch, where clusters either contain sequences from the same line or alternatively from a number of different parasites. The second large group containing the remaining two-thirds of *stevor *sequences appears to contain numerous branch points of related sequences rather than containing a single defined cluster. These data would indicate that overall the sequence divergence is much higher in this group. Sequences obtained from a single parasite line also appear to form distinct clusters, with each cluster containing a number of different related sequences. This can be clearly seen for T9/96 *stevor *(Figure 3A, blue circles) that contains at least three distinct clusters. Similar clustering is also seen for sequences from A4, C10, FcB1 and D10 with the majority of sequences falling within just four sub-groups (Figure 3A, green circles) while the majority of Kilifi isolates *stevor *sequences were contained within seven clusters (Figure 3A, yellow circles). Importantly, these specific sequence clusters often also contain sequences from other parasite lines or isolates indicating that they do not result from a specific phylogenetic branch. Rather it appears that while there is a trend for some sequences from the same parasite to cluster together, most are distributed across the tree and cluster with sequences from other strains. To further validate these findings, the *stevor *sequences were clustered using the Minimum Evolution tree construction method at 95% significance bootstrap values (Figure 3B). This approach produced the same clusters as the Neighbor Joining method (compare Figure 3A with 3B). The 3D7 *stevor *repertoire is distributed throughout the trees (Figure 3A, B; pink points) with clusters containing a maximum of two 3D7 sequences.

**Figure 4:**
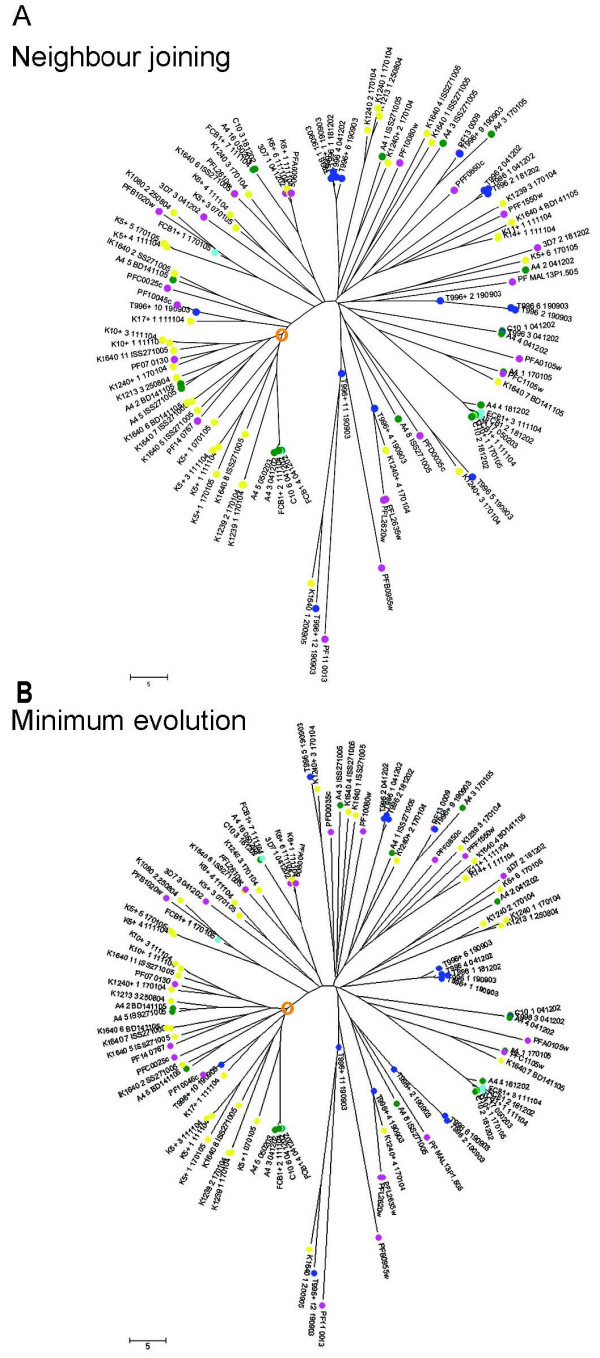
**Phylogenetic trees of *stevor *HVR protein sequences**. Unrooted phylogenetic trees using neighbor-joining and minimum evolution methods showing all branches (A) and 85% condensed trees (B) using 108 amino acid sequences. Trees are based upon an alignment of the HVRs then condensed to show only those branches with a bootstrap value of 85% significance and above.

To assess whether or not this clustering of *stevor *DNA sequences is maintained at the amino acid level, 108 unique STEVOR amino acid sequences were used to construct a Neighbor Joining as well as a Minimum Evolution Tree with a bootstrap value of 85% or higher (Figure 4A, B). The two approaches resulted in nearly identical trees with a number of broad clusters containing sequences from different lines and isolates. Using this analysis a single major sub-group was observed (highlighted by an orange circle) reflecting an apparent trend for sequences of the same geographic region to be more closely related (Figure 4A, B). More precisely, one obvious cluster was observed containing majority of Kilifi protein sequences (Figure 4A, orange circle, yellow points). However, as already identified in the DNA sequence trees, clusters are not completely exclusive and a number of sequences from laboratory lines are found within this cluster (Figure 4A, orange circle, green, pink, blue and light blue points). Conversely a number of Kilifi isolate sequences are found distributed across the other branches of the tree where they cluster both with other Kilifi sequences as well as sequences from a range of laboratory lines (Figure 4, yellow points).

Similar to the outcome of the DNA sequence analysis, the T9/96 amino acid sequences clustered within three small sub-groups (Figure 4, blue circles) while sequences from laboratory lines A4 (green), C10 (green), FcB1 (light blue) could be found dispersed in different clusters on the tree (Figure 4). Interestingly, the significant clustering seen within the trees produced from the nucleotide sequences (Figure 3) was also maintained at the amino acid level. This clustering is consistent using either of two methods for generating the phylogenetic trees.

**Figure 5:**
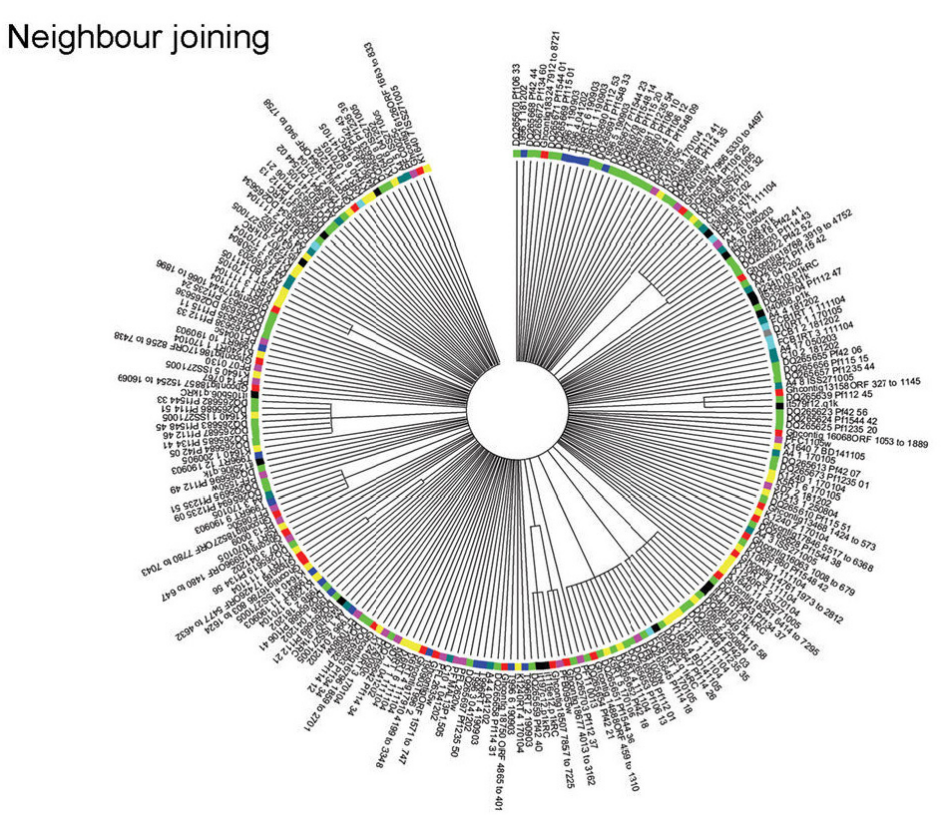
**50% condensed tree using 226 amino acid sequences**. Sequences are color-coded according to originating isolate: Kilifi field isolates-yellow; T9/96-dark blue; 3D7-pink; A4 and C10-green; FcB1-light blue; D10-light green, IT-black, Ghanaian field isolate-red. Orange open-circle indicates a major *stevor *sub-group, blue open-circles highlight T9/96 sub-groups, green open-circles highlight sub-groupings including FcB1, C10 and A4 sequences, yellow open-circles highlight Kilifi sequence sub-groupings.

This analysis was further extended to include all STEVOR sequences including those from published databases (IT and Ghanaian isolates) and 75 South American region sequences obtained from Albrecht *et al*. (2006)[18]. A total of 226 HVR amino acid sequences were used for the analysis (Figure 5). However, no obvious cluster was observed, though a number of non-significant groups were present when the tree was condensed at 50% bootstrap values. The high divergence within the HVR encoding the predicted erythrocyte surface-exposed region of the STEVOR family is likely in response to host immunity.

## Discussion

The human malaria parasite *P. falciparum *is able to evade the host's immune response by expressing a range of variable antigens on the surface of the infected erythrocyte. Several genes are potentially involved in this process with the *var*, *rif *and *stevor *[2] multigene families being the most likely candidates and coding for rapidly evolving proteins. The rapid changes are believed to result from a strong immune-mediated diversifying selection [19,43,44].

In this study, the *stevor *sequence repertoire and diversity were analysed in laboratory lines and Kilifi field isolates. As expected the *stevor *family was found in all *P. falciparum *genomes and was transcribed in all field and laboratory parasites (exception for the A4 clone) with multiple transcripts being detected. This result is in line with previous studies in which numerous *var *as well as *stevor *transcripts were detected in single patient samples using a microarray [45]. From the RT-PCR and sequencing data it is clear that both in cultured laboratory lines and in patient samples a significant proportion of the *stevor *repertoire is transcribed at the same time. In isolate K5 a total of 16 different sequences were detected; and despite the close phylogenetic relationship between these sequences (Figure 3) at this stage it can not be ruled out that these transcripts arose from a multiclonal infection.

Recent phylogenetic studies have shown that the *rif *family form two clear subgroups [24] that potentially can be further divided into functionally distinct subgroups [46]. This is clearly not the case for *stevor *where the presence of distinct subgroups is not that strongly supported. Still there is sufficient evidence to support some clustering into different groups. Importantly, the identification of identical sequences in laboratory lines from different geographic regions as well as field samples would imply some functional constraints acting upon this sequence. In addition sequences within the Kilifi isolates appear to be more closely related to each other than to any of the laboratory parasites, and no common *stevor *were found between Kilifi except for a single *stevor *sequence shared between one Kilifi isolate and 3D7. The *stevor *sequence found in two Kilifi isolates (K11 and K14) may represent a common sequence detected in the two isolates and also seen in a number of laboratory lines.

## Conclusion

Conserved genes were identified in different *P. falciparum *isolates from different global locations for example: A4, C10 and FcB1 in particular, contain several closely related *stevor *genes, suggesting that the ancestral *P. falciparum *parasite genome already had multiple *stevor *genes that may have subsequently diversified further within the different *P. falciparum *populations. The high sequence variability observed is consistent with the HVR of STEVOR being under strong selection pressure. On the other hand, there is only weak evidence that STEVOR like the RIFIN can be subdivided into distinct phylogenetic subgroups implying that STEVOR function has less diversified in *P. falciparum*.

## Competing interests

The authors declare that they have no competing interests.

## Authors' contributions

JEB carried out molecular biology studies and wrote the paper. MN analysed the data and wrote the paper. AAH, KM and JL participated in the design and coordination of the study and helped to draft the manuscript. PRP conceived the study, and participated in its design and coordination and wrote the paper. All authors read and approved the final manuscript.

## Supplementary Material

Additional file 1 **Stevor DNA sequences**. The data provided represent nucleotide sequences of clones obtained from PCR and RT-PCR reactions of lab strains and field isolates. Click here for file
